# β_2_-Agonists Inhibit TNF-α-Induced ICAM-1 Expression in Human Airway Parasympathetic Neurons

**DOI:** 10.1371/journal.pone.0044780

**Published:** 2012-09-25

**Authors:** Zhenying Nie, Allison D. Fryer, David B. Jacoby

**Affiliations:** Division of Pulmonary and Critical Care Medicine, Medical School Oregon Health and Science University, Portland, Oregon, United States of America; University of California, Merced, United States of America

## Abstract

**Background:**

Major basic protein released from eosinophils to airway parasympathetic nerves blocks inhibitory M_2_ muscarinic receptors on the parasympathetic nerves, increasing acetylcholine release and potentiating reflex bronchoconstriction. Recruitment of eosinophils to airway parasympathetic neurons requires neural expression of both intercellular adhesion molecular-1 (ICAM-1) and eotaxin. We have shown that inflammatory cytokines induce eotaxin and ICAM-1 expression in parasympathetic neurons.

**Objective:**

To test whether the β_2_ agonist albuterol, which is used to treat asthma, changes TNF-alpha-induced eotaxin and ICAM-1 expression in human parasympathetic neurons.

**Methods:**

Parasympathetic neurons were isolated from human tracheas and grown in serum-free medium for one week. Cells were incubated with either (R)-albuterol (the active isomer), (S)-albuterol (the inactive isomer) or (R,S)-albuterol for 90 minutes before adding 2 ng/ml TNF-alpha for another 4 hours (for mRNA) or 24 hours (for protein).

**Results and Conclusions:**

Baseline expression of eotaxin and ICAM-1 were not changed by any isomer of albuterol as measured by real time RT-PCR. TNF-alpha induced ICAM-1 expression was significantly inhibited by (R)-albuterol in a dose dependent manner, but not by (S) or (R,S)-albuterol. Eotaxin expression was not changed by TNF-alpha or by any isomer of albuterol. The β-receptor antagonist propranolol blocked the inhibitory effect of (R)-albuterol on TNF-alpha-induced ICAM-1 expression.

**Clinical Implication:**

The suppressive effect of (R)-albuterol on neural ICAM-1 expression may be an additional mechanism for decreasing bronchoconstriction, since it would decrease eosinophil recruitment to the airway nerves.

## Introduction

Eosinophils are in contact with airway nerves in patients with asthma and in antigen challenged animals [1], [2]. Migration and binding of eosinophils to the nerve are mediated by chemotactic factors and adhesion molecules [3], [4], [5], [6], [7], [8], [9], including eotaxin and intercellular adhesion molecule 1 (ICAM-1). Eotaxin selectively recruits eosinophils via CCR3 (C–C chemokine receptor 3) expressed on eosinophils. ICAM-1 is important for eosinophil adhesion via LFA-1, a receptor found on eosinophils. Both eotaxin and ICAM-1 are present on airway nerves in antigen-challenged guinea pigs and on cultured airway parasympathetic neurons [5], [7]. Both can be induced by inflammatory cytokines [5], [7], [10], [11]. Reducing ICAM-1 or blocking eotaxin expression on parasympathetic nerves relates to reduced parasympathetic nerves associated eosinophils, and reduced airway hyperreactivity [5], [7], [8]. Thus, controlling expression of eotaxin and ICAM-1 on airway parasympathetic nerves is critical for reducing neural inflammation and preventing airway hyperreactivity.

The short-acting β_2_-adrenergic bronchodilator albuterol is commonly administered to patients in racemic form, containing equal parts of its active isomer (R)- and its inactive isomer (S)-albuterol. It has been argued that (R)-albuterol (often known as levalbuterol) is more effective than the racemic (R, S)-albuterol mixture. Clinical studies demonstrate that greater clinical efficacy is achieved when (R)-albuterol is given in amounts equivalent to that found in the racemic albuterol and that (R)-albuterol is also associated with fewer side effects [12], [13]. The mechanism underlying the difference between (R)- and (R, S)- albuterol remains unclear. Since the expression of eotaxin and ICAM-1 on airway parasympathetic nerves are critical for neural inflammation, we tested the effect of (R,S)-albuterol, (R)-albuterol and (S)-albuterol on TNFα-induced eotaxin and ICAM-1 expression on human parasympathetic neurons in primary culture.

## Results

### β_2_ Receptors are Expressed on Human Parasympathetic Neurons

**Figure 1:**
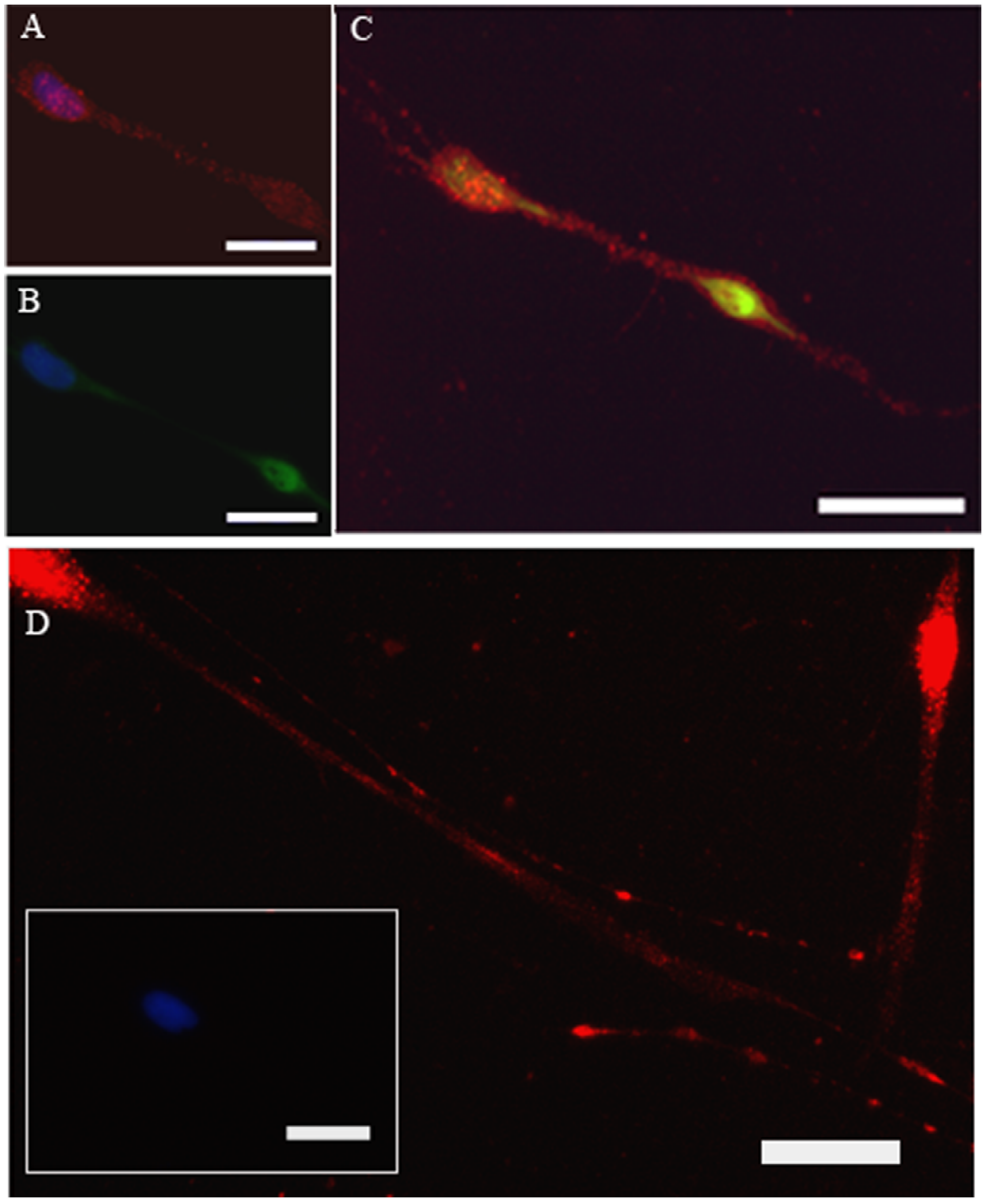
β_2_ receptors are identified by anti-β_2_ receptors antibody on human trachea parasympathetic neurons (red, A, B–D) under high (A,C) and low (D) power. Neurons are labeled with anti-neurofilament antibodis (B, green) and the merged image (for neuronal and β_2_ receptor staining) is shown in C. Nuclei stain blue with DAPI. The insert of D is the absence of primary antibody. Magnification bars: 50 µm.

β_2_ receptor expression was shown by staining with anti-β_2_ receptor antibody (red, Figure 1 A and C–D). Parasympathetic neurons were identified in primary culture using antibodies to non-phosphorylated neurophilaments (green, Figure 1 B–C). Parasympathetic neurons expressed β_2_ receptors as shown by positive co-localization (yellow, Figure 1C) of anti-β_2_ receptor (red) and anti-neurophilament (green) antibodies staining. β_2_ receptors were expressed on the cell body (Figure 1 A,C–D) and neurites (Figure 1 D). There was no fluorescent signal in negative controls (insert of D) that were treated with normal serum in place of primary antibodies. Cell nuclei were stained blue with DAPI. (Figure 1 A–C and insert of D).

### Different Effects on TNF-α Induced ICAM-1 and Eotaxin Expression by Different Albuterol Isomers

**Figure 2:**
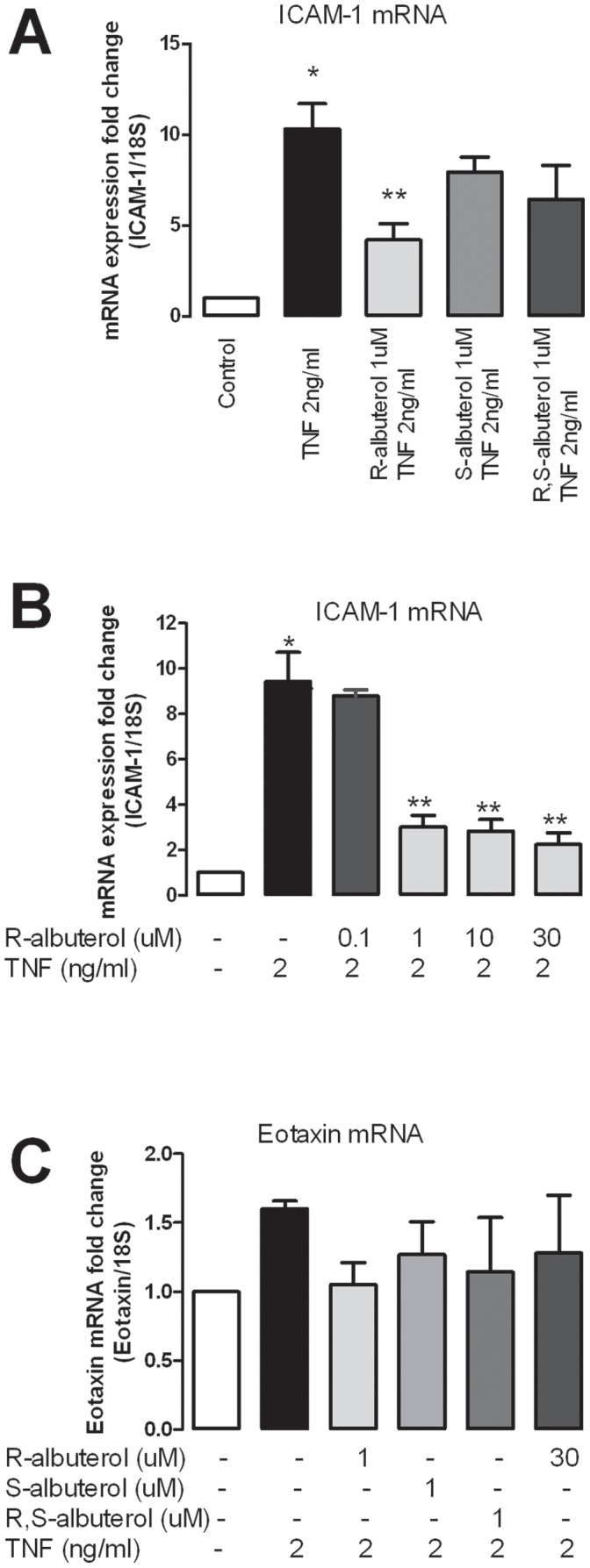
Pretreatment with (R)-albuterol before TNF-α significantly inhibits TNF-α-induced ICAM-1 mRNA expression in human parasympathetic neurons as detected by real-time qPCR (A). (S)-or (R,S)-albuterol does not inhibit TNF-α induced ICAM-1 (A). The inhibitory effect of (R)-albuterol on TNF-α-induced ICAM-1 mRNA expression is dose dependent (B). Neither TNF-α nor any albuterol isomer changes eotaxin expression (C). *indicates significantly different from control. **indicates significantly different from TNF-α treatment, as analyzed by one way ANOVA.

The anti-inflammatory effect of albuterol was tested by investigating the effect of albuterol on TNF-α-induced ICAM-1 and eotaxin mRNA expression (Figure 2). TNF-α significantly induced ICAM-1 mRNA expression on human parasympathetic neurons (Figure 2A) as compared to control.

(S)-, and (R,S)-albuterol (1 uM) caused a small but not statistically significant decrease in TNF-α-induced ICAM-1 expression (P>0.05) (Figure 2A). In contrast, (R)-albuterol (1 uM) significantly inhibited TNF-α-induced ICAM-1 mRNA expression by more than 50% (P<0.05, Figure 2A). The inhibitory effect of (R)-albuterol on TNF-α-induced ICAM-1 expression was dose dependent (Figure 2B). None of the isomers of albuterol changed basal mRNA expression of ICAM-1 in human parasympathetic neurons (data not shown). Neither albuterol nor TNF-α changed the expression of eotaxin (P>0.05, Figure 2C).

**Figure 3:**
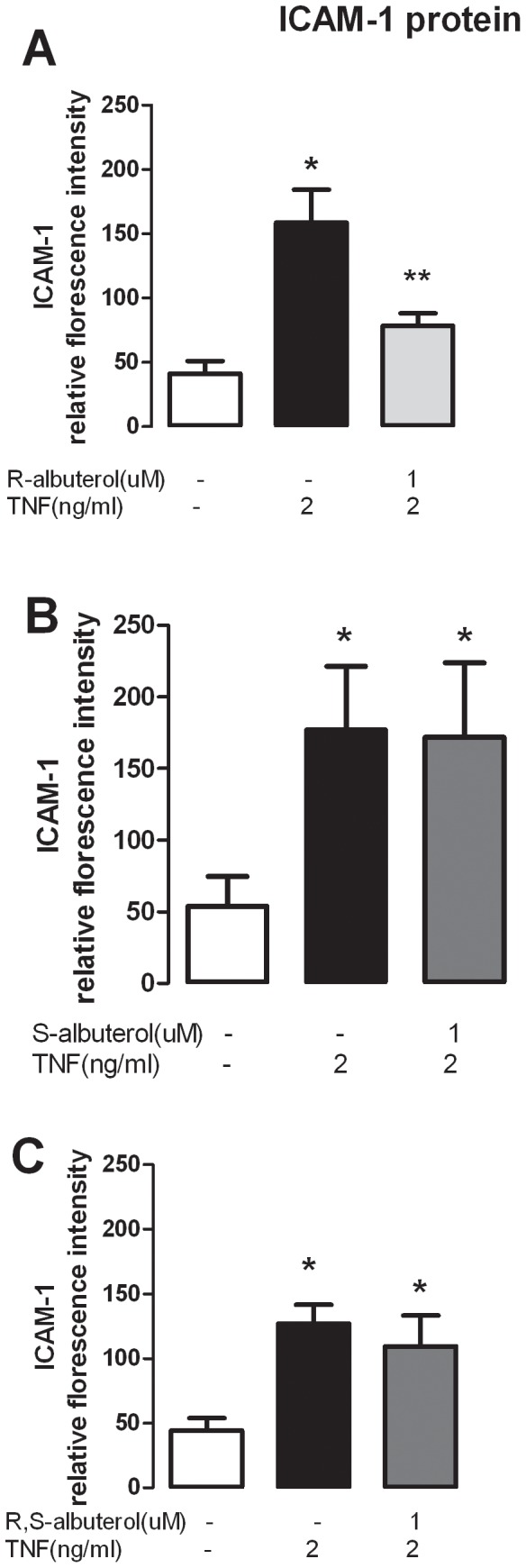
ICAM-1 protein expression is measured by fluorescence intensity of a labeled anti-ICAM-1 antibody. (R)-albuterol (A) but not S- or (R-,S)-albuterol (B and C) significantly inhibits TNF-α-induced ICAM-1 protein (p<0.005). *indicates significantly different from control and **indicates significantly different from TNF- treatment, as analyzed by paired T-test.

The effect of albuterol on TNF-α-induced ICAM-1 protein expression was tested by measuring the fluorescence intensity of immunohistochemical staining (Figure 3). Consistent with our previous finding [5], [7], TNF-α significantly increased ICAM-1 protein expression in human parasympathetic neurons (Figure 3). (R)-albuterol significantly inhibited TNF-α-induced ICAM-1 protein expression (Figure 3A; p<0.05). In contrast, neither (S) nor (R,S)-albuterol affected TNF-α-induced ICAM-1 protein expression (Figure 3 B and C).

**Figure 4:**
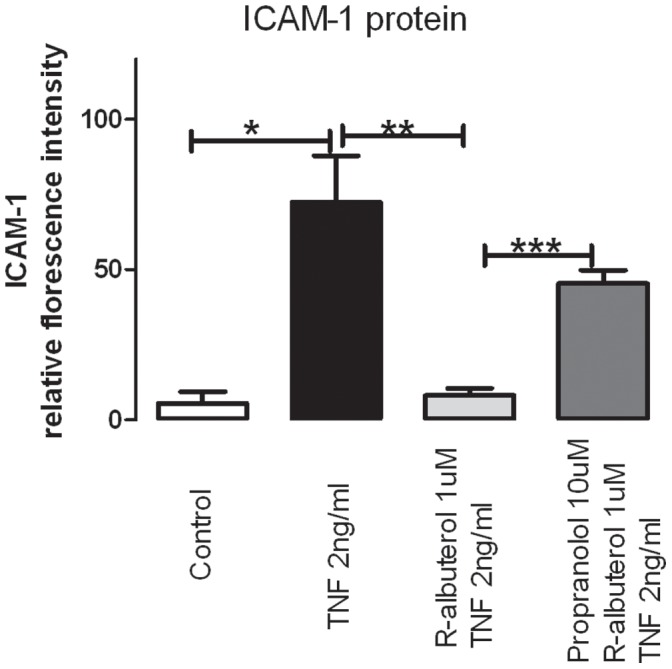
Pretreatment with β-receptor antagonist propranolol completely prevents the suppressive effect of R-albuterol on TNF-α-induced ICAM-1 protein expression that is identified by fluorescence intensity of anti-ICAM-1 antibody staining in human parasympathetic nerves. *indicates significant difference from control, ** indicates significant difference from TNF-α treatment and *** indicates significant difference from (R)-albuterol, as analyzed by one way ANOVA.

In order to test whether the inhibitory effect by R-albuterol was via β-receptors, human parasympathetic neurons were pre-incubated with and without the β-receptor antagonist propranolol before (R)-albuterol and TNF-α were applied to the cell culture (Figure 4). In neurons pre-incubated with propranolol and (R)-albuterol, ICAM-1 protein expression induced by TNF-α was significantly (6 times) higher than those with (R)-albuterol but without propranolol pre-incubation (p<0.05) (Figure 4). Additionally, ICAM-1 expression in the propranolol and (R)-albuterol pre-incubation group was not significantly different from that treated with TNF-α and propranolol (Figure 4). Thus, the suppression effect of albuterol was blocked by pre-incubation with propranolol, indicating that (R)-albuterol inhibits TNF-α induced ICAM-1 expression on human parasympathetic neurons via β-receptors.

## Discussion

The data presented here are the first to directly show expression of β_2_-receptors on parasympathetic neurons (Figure 1), although it has been investigated indirectly in physiology and pharmacology studies [14], [15], [16]. Our data show that activation of β_2_-receptors by (R)-albuterol, but not (S)- or (R,S)- albuterol, can inhibit TNF-α-induced ICAM-1 expression on parasympathetic neurons. Reduced ICAM-1 expression on parasympathetic nerves is directly related to reduced interaction between parasympathetic nerves and inflammatory cells, including eosinophils [7], [8]. It is known that inflammatory cells can affect the release of neurotransmitters from nerves. For example, eosinophils increase ACh release, leading to airway hyperreactivity [2], [5], [7], [8], [17], [18]. Thus, the inhibitory effect of R-albuterol on TNF-α-induced ICAM-1 expression can reduce recruitment of inflammatory cells to the parasympathetic nerve, inhibiting airway hyperreactivity.

β_2_-agonists have several non-bronchodilator actions that may contribute to their clinical efficacy. For example, β_2_-agonists have inhibitory effects on inflammatory cells, including neutrophils [19], T lymphocytes [20], and eosinophils [21]. β_2_-agonists can also down regulate eotaxin production in airway smooth muscle cells [22]. The observation presented here provides another piece of evidence supporting the anti-inflammatory effect of β_2_-adrenergic agonists (Figure 2–4). Because airway parasympathetic nerve associated eosinophils play a key role in antigen induced hyperreactivity and the migration of eosinophils is mediated by ICAM-1 [5], [7], [8], [23], [24], [25], the suppressing effect of (R)-albuterol on neuronal ICAM-1 expression may be an important anti-inflammatory effect of this drug which contribute to its clinical efficacy.

(R)-albuterol is more potent in inhibiting TNF-α-induced ICAM-1 expression than (S)-albuterol or (R,S)-albuterol. (S)-albuterol does not contribute to the anti-inflammatory effects (Figure 2 and 3), but may compete with (R)-albuterol for binding to β_2_-receptor in the racemic albuterol ((R,S)-albuterol) as (S)-albuterol is a partial agonist at the β_2_-receptor [26]. Moreover, at the same concentration of albuterol, racemic albuterol ((R,S)-albuterol) contains only half of the albuterol that can efficiently bind to β_2_-receptors and inhibit TNF-α-induced ICAM-1 expression. Therefore, it is not surprising that (R)-albuterol is more potent compared to (R,S)-albuterol (Figure 2 and 3) in inhibiting ICAM-1 expression on parasympathetic neurons.

Our previous study also shows that the NF-κB inhibitor blocks TNF-α-induced ICAM-1 expression, indicating that TNF-α induces ICAM-1 expression on parasympathetic neuron via a NF-κB dependent mechanism [7]. This is consistent with results from studies in other type of cells, such as airway epithelial cells [27], that NF-κB is a key player in ICAM-1 expression induced by TNF-α. Therefore, factors that influence the translocation and activation of NF-κB proteins may change TNF-α-induced ICAM-1 expression. An inhibitory effect of the β_2_-receptor agonist on NF-κB activity has been reported [28]. β_2_-receptor agonists regulate NF-κB activity via elevated cAMP and activation of PKA [28], [29], [30], [31]. This suggests that R-albuterol, a β_2_-receptor agonist, suppresses the activity of NF-κB via elevated cAMP and activation of PKA, inhibiting TNF-α-induced expression of ICAM-1 mRNA.

In summary, we have shown that β_2_-adrenergic receptors are expressed on human parasympathetic neurons and activation of β_2_-adrenergic receptors by (R)-albuterol can suppress TNF-α-induced ICAM-1 expression. As this effect is not seen with racemic albuterol, we speculate that (R)-albuterol may have a better therapeutic effect than racemic albuterol in asthma treatment, not only because of its bronchodilator properties, but also because of its anti-inflammatory effect.

## Materials and Methods

Cell culture: Airway parasympathetic neurons were isolated from human tracheas which were donated by organ donors. The Oregon Health & Science University Research Integrity Office waived the need for ethical approval on this culture of human airway cells prior to the onset of the study. Airway parasympathetic neurons were grown in serum-free medium for 1 week as previously described [7]. Cells were incubated in fresh medium with or without albuterol isoforms (Sepracor) for 90 minutes, followed by additional incubation with recombinant human TNF-α (2 ng/ml, T0157 Sigma) for either 4 hours for real time RT-PCR, or 24 hours for immunohistochemistry. In experiments with propranolol (10 uM, Sigma P0884), the β-blocker was added to the culture medium 30 minutes before (R)-albuterol was applied.

Immunohistochemistry staining: Parasympathetic neurons were identified by staining with antibodies to non-phosphorylated neurophilament (SMI-311, 1∶10000, Covance). β_2_ receptors were detected using rabbit anti-β_2_ receptor antibody (1∶200, sc-569, Santa Cruz Biotechnology, Inc., Santa Cruz, CA). ICAM-1 expression in parasympathetic nerves from human tracheas was identified using rabbit anti-human ICAM-1 antibodies (1∶50, sc-7891, Santa Cruz Biotechnology, Inc., Santa Cruz, CA). All primary antibodies were incubated overnight at 4°C, followed by incubation with corresponding secondary antibodies, either labeled with Alexa fluor 555 (red) or Alexa fluor 488 (green) (Molecular Probes, Invitrogen Corp., Carlsbad, CA ) for 60 minutes at 37°C. Negative controls were incubated with normal serum (Vector) in place of the primary antibodies. All slides were mounted in aqueous medium with 4′-6′diamino-2-phenylindole (DAPI, Vector) to stain nuclei.

Florescence intensity of ICAM-1 staining was quantified using Metamorph. Cells, which were cultured on Lab Tek IV chamber slides (cat#154461, Nalge Nunc International), were divided into groups as follows: control, TNF-α treated only, albuterol isomer plus TNF-α treated, and propranolol plus R-albuterol plus TNF-α treated. The labeling of each slide was covered in order to eliminate investigator bias. All chamber slides were treated the same way in each step during the fixation and immunohistochemical staining and were photographed using a fluorescence microscope with the same exposure time. ICAM-1 labeled neurites from different treatment groups were selected at random and outlined. Nerve cell bodies were difficult to distinguish individually and therefore were not included in the analysis. The lower threshold, set as zero, was determined in empty areas using Metamorph, and was then used to measure the intensity in neurites. Average intensity was collected from twenty to thirty separate neurites in each treatment group and the experiment was repeated three or more times. The mean ± standard error was calculated from collected data and one way ANOVA test was used to determine the statistical significance. *P* values of less than 0.05 were accepted as statistically significant.

Real time RT-PCR: Cell RNA was isolated using the RNeasy Mini Kit (74106; QIAGEN), according to the manufacturer’s recommendations. The RNA was reverse transcribed using SuperScript III (18080–051; Invitrogen Corp.) with random hexamer primers. Quantitative PCR was carried out in triplicate at 60°C over 45 cycles using the Quantitect SYBR Green PCR kit (204143; QIAGEN). The amounts of PCR product were quantified using the Mx3000P real-time PCR system (Stratagene). Oligonucleotide PCR primer pairs were designed from published human sequences as follows*:* ICAM-1∶5′-GGCTGGAGCTGTTTGAGAAC-3′ and 5′-ACTGTGGGGTTCAACCTCTG-3′. eotaxin-1: sense, AACCACCTGCTGCTTTAACC, antisense, TCCTGCACCCACTTCTTCTT. 18S ribosomal RNA was used as an internal control, and the primer pairs were as follows: 5′-GTAACCCGTTGAACCCCATT-3′ and 5′-CCATCCAATCGGTAGTAGCG-3′. The threshold cycle number was measured, and the relative expression of ICAM-1 was adjusted for the threshold cycle for detection of 18S. Results were presented as the mean ± SE of the 3 experiments from human parasympathetic neurons isolated from the trachea. A one way ANOVA was used to determine the statistical significance of differences. *P* values of less than 0.05 were accepted as statistically significant.
